# Retrospective study of canine endoparasites diagnosed by fecal flotation methods analyzed across veterinary parasitology diagnostic laboratories, United States, 2018

**DOI:** 10.1186/s13071-021-04960-7

**Published:** 2021-08-31

**Authors:** Caroline Sobotyk, Kaitlyn E. Upton, Manigandan Lejeune, Thomas J. Nolan, Antoinette E. Marsh, Brian H. Herrin, Mindy M. Borst, Julie Piccione, Anne M. Zajac, Lauren E. Camp, Cassan N. Pulaski, Lindsay A. Starkey, Cristiano von Simson, Guilherme G. Verocai

**Affiliations:** 1 Department of Veterinary Pathobiology, College of Veterinary Medicine and Biomedical Sciences, Texas A&M University, College Station, TX 77843 USA; 2 Department of Population Medicine and Diagnostic Sciences, Cornell University College of Veterinary Medicine, Ithaca, NY 14850 USA; 3 Department of Pathobiology, University of Pennsylvania, School of Veterinary Medicine, Philadelphia, PA 19104 USA; 4 Department of Veterinary Preventive Medicine, College of Veterinary Medicine, The Ohio State University, Columbus, OH 43210 USA; 5 Department of Diagnostic Medicine and Pathobiology, College of Veterinary Medicine, Kansas State University, Manhattan, KS 66506 USA; 6 Texas A&M Veterinary Medicine Diagnostic Laboratory, Texas A&M University, College Station, TX 77841 USA; 7 Department of Biomedical Sciences and Pathobiology, Virginia Tech, Blacksburg, VA 24061 USA; 8 Veterinary Medical Teaching Hospital, University of California, Davis, CA 95616 USA; 9 Department of Infectious Diseases, University of Georgia College of Veterinary Medicine, Athens, GA 30602 USA; 10 Department of Pathobiology, College of Veterinary Medicine, Auburn University, Auburn, AL 36849 USA; 11 Virbac Animal Health, Fort Worth, TX USA

**Keywords:** Diagnostics, Dog, Endoparasitism, Fecal flotation, Helminths, Protozoa, Zoonosis

## Abstract

**Background:**

Companion animal endoparasites play a substantial role in both veterinary medicine and public health. Updated epidemiological studies are necessary to identify trends in occurrence and distribution of these parasites, and their associated risk factors. This study aimed to assess the occurrence of canine endoparasites  retrospectively, using fecal flotation  test data available through participating academic veterinary parasitology diagnostic laboratories across the United States of America (USA).

**Methods:**

Canine fecal flotation records from ten veterinary diagnostic laboratories located in nine states in the USA acquired from January 1, 2018, to December 31, 2018, were included.

**Results:**

A total of 4692 fecal flotation test results were obtained, with a majority comprised of client-owned dogs (3262; 69.52%), followed by research dogs (375; 8.00%), and shelter dogs (122; 2.60%). Samples from 976 (20.80%) dogs were positive for at least one parasite, and co-infections of two or more parasites were found in 3.82% (179/4692) of the samples. The five most commonly detected parasites were: *Giardia* sp., (8.33%; 391/4692), Ancylostomatidae (5.63%; 264/4692), *Cystoisospora* spp. (4.35%; 204/4692), *Toxocara canis* (2.49%;117/4692), and *Trichuris vulpis* (2.43%; 114/4692). Various other internal parasites, including gastrointestinal and respiratory nematodes, cestodes, trematodes, and protozoans were detected in less than 1% of samples.

**Conclusions:**

These data illustrate the importance of parasite prevention, routine fecal screening, and treatment of pet dogs. Additionally, pet owners should be educated about general parasite prevalence, prevention, and anthelmintic treatment regimens to reduce the risks of environmental contamination and zoonotic transmission.

**Graphical Abstract:**

**Figure:**
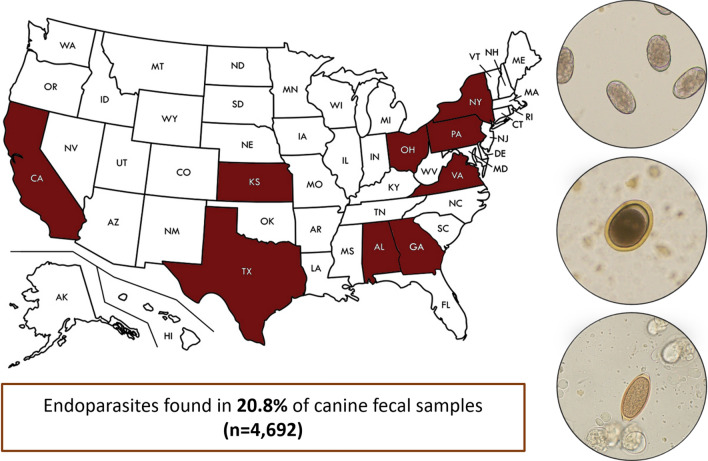

**Supplementary Information:**

The online version contains supplementary material available at 10.1186/s13071-021-04960-7.

## Background

Companion animals are hosts for a variety of endoparasites of veterinary and public health importance, including gastrointestinal (GI) helminths and protozoans. The prevalence of various canine endoparasites across the United States of America (USA) depends on geographic location, dog population being studied, and diagnostic techniques used for sample analysis [1–3]. Among the most commonly detected endoparasites of dogs across most of the world, including North America, are GI nematodes such as *Ancylostoma caninum, Toxocara canis*, and *Trichuris vulpis*, and the flagellate protozoan *Giardia* sp. [2, 4, 5]. Due to close contact between dogs and humans, the zoonotic potential of canine GI parasites has been extensively investigated in different geographic locations [6–8]. This fact highlights the importance of both monitoring parasite distribution and evaluating parasitic infections of pet dogs.

Although subclinical infections are common, clinical disease and varied clinical signs may be associated with GI parasite infections in companion animals including diarrhea, vomiting, weight loss, loss of appetite, and anemia [4]. The frequency and severity of clinical disease varies greatly with the parasite species involved, host age, nutritional condition, and immunity [7, 9]. However, infected dogs with no clinical signs remain an important contributor and relevant risk factor for environmental contamination and further transmission to other susceptible hosts [4, 10, 11]. Therefore, annual fecal analysis for GI parasite infections should be standard even in dogs showing no clinical signs and/or on routine parasite preventive products [12]. Several methods are available to diagnose parasitic infections in companion animals. The sensitivity and limitations of each technique vary according to the parasite species, sample analyzed, and the technique itself [13, 14]. For GI parasites, fecal flotation techniques are the most frequently used method for detection of diagnostic parasite stages such as helminth eggs and protozoan cysts or oocysts in both clinical settings and diagnostic laboratories. Generally, flotation methods involving double or single centrifugation are recommended because these are more sensitive than passive gravitational flotation techniques in recovering parasite diagnostic stages [3, 15, 16]. Nevertheless, an accurate diagnosis is crucial for clinical decision-making, including appropriate control and prevention measures.

Despite current recommendations for broad-spectrum parasite prevention protocols and routine diagnostics, endoparasites still pose a risk to public health. Updated epidemiological studies, including those using retrospective data, are valuable to identify trends in the occurrence and distribution of companion animal parasites, given their importance in veterinary and public health. This study aimed to assess the occurrence of canine endoparasites  using retrospective  fecal flotation  data available through academic veterinary parasitology diagnostic service laboratories across the USA in the calendar year 2018.

## Methods

We performed a retrospective study of GI parasite infections in dogs using records of fecal flotation analyses from veterinary diagnostic service laboratories across the USA during a 1-year period from January 1, 2018, to December 31, 2018. Test results were acquired from ten diagnostic laboratories across nine states, including Alabama, California, Georgia, Kansas, New York, Ohio, Pennsylvania, Texas, and Virginia.

The data collected were entered into an Excel spreadsheet, and compiled by personnel at Texas A&M University. Results of all fecal flotation tests using both single and double centrifugation methods, with sodium nitrate, zinc sulfate, and/or Sheather’s sucrose as flotation solutions were included in this study. Results from direct smear, sedimentation, and Baermann tests were excluded as they were available for only a small number of samples. The fecal analyses, interpretation of results, and parasite identifications were performed by trained laboratory personnel according to each laboratory’s standard operating procedures and based on microscopic assessment of morphological features of parasite diagnostic stages.

When available, the following information was included in the datasheet for each dog: identification number, location, origin, breed, age, sex, reproductive status, and month of fecal exam. For analysis of age, data were grouped into < 1 year (young), 1–6 years (adult), and > 6 years (senior). Breeds were grouped as working, herding, hound, non-sporting, sporting, toy, terrier, and foundation stock service dogs according to the American Kennel Club [17]. Mixed-breed dogs were those designated in the database as mixed-breed or as belonging to a breed not recognized by the American Kennel Club [17]. Origin was grouped as teaching hospital, outside practitioner, research, shelter, and referral laboratory according to the location where the animal was presented and/or fecal sample collected. Multiple fecal floatation analyses for the same sample performed in the same period were considered a single result. A Chi-square test was performed to determine the significance of breed, sex, age of the animals, geographic location, and season of the year on parasite occurrence. Results were considered significant when *p* < 0.05. Occurrence and 95% confidence intervals (CI) were determined for each variable.

## Results

Results of 4692 fecal flotation tests were obtained from electronic medical records and included in this study. Overall, 976 (20.8%) canine fecal samples tested positive for at least one parasite. Single infections were recorded in 16.99% (797/4692) of samples, while co-infections with two or more endoparasites were recorded in 3.82% (179/4692) (Table 1). The occurrence of co-infections was significantly higher in dogs under 1 year old (22.58%; 91/403) when compared to adult and senior animals. Protozoans were detected most often with an occurrence of 13.36% (627/4692), followed by nematodes 11.25% (528/4692), cestodes 0.45% (21/4692), and trematodes 0.09% (4/4692). The most commonly detected parasite was *Giardia* sp., which was detected in 8.33% (391/4692) of the dogs, followed by Ancylostomatidae 5.63% (264/4692), and *Cystoisospora* spp. 4.35% (204/4692). *Cystoisospora* spp. consisted of the combined reports of *C. canis* (0.96%, 45/4692), *C. ohioensis*-complex (2.22%, 104/4692), and *Cystoisospora* sp. (1.17%, 55/4692). *Toxocara canis* had an occurrence of 2.49% (117/4692) followed by *T. vulpis* with 2.43% (114/4692). Other GI parasites detected in less than 1% of samples included the protozoans *Sarcocystis* sp.*,* and *Neospora caninum*/*Hammondia*; the nematodes *Eucoleus boehmi*, *Toxascaris leonina*, *Eucoleus aerophilus,* and *Uncinaria stenocephala*; the cestodes *Dipylidium caninum* and Taeniidae. Although fecal flotation is not considered gold standard method for detecting several protozoan oocysts, nematode larvae, and trematode eggs, some endoparasites were also found in a small number of samples, including oocysts of *Cryptosporidium* sp.; first-stage larvae of *Strongyloides stercoralis* and *Crenosoma* sp.; and eggs of *Physaloptera* sp., *Pearsonema* sp., *Spirometra* sp., *Alaria* sp., *Heterobilharzia americana*, *Nanophyetus salmincola*, and *Paragonimus kellicotti* (Table 2)*.* Spurious parasites were detected in 5.67% (266/4692) of samples, including *Eimeria* sp., *Monocystis* sp., and *Toxocara cati*.

**Table 1:** Prevalence of single and co-infections by endoparasites among dogs (n = 4692) in the US

Parasite infection	Positives (n)	Prevalence % (95% CI)
Single infections	797	16.99 (15.91–18.06)
Co-infections	179	3.82 (3.27–4.36)
Co-infection with 2 parasites	155	3.30 (2.79–3.81)
Co-infection with 3 parasites	22	0.47 (0.27–0.66)
Co-infection with 4 parasites	1	0.02 (0–0.06)
Co-infection with 5 parasites	1	0.02 (0–0.06)

**Table 2:** Prevalence of different endoparasites among dogs (n = 4692) in the US

Parasite infection	Positives (n)	Prevalence % (95% CI)
Helminths		
Nematodes		
Ancylostomatidae^a^	264	5.63 (4.97–6.29)
* Toxocara canis*	117	2.49 (2.05–2.94)
* Trichuris vulpis*	114	2.43 (1.99–2.87)
* Eucoleus boehmi*	14	0.30 (0.14–0.45)
* Strongyloides stercoralis*	8	0.17 (0.05–0.29)
* Toxascaris leonina*	4	0.09 (0–0.17)
* Eucoleus aerophilus*	2	0.04 (0–0.10)
*Physaloptera* sp.	2	0.04 (0–0.10)
* Crenosoma*	1	0.02 (0–0.06)
* Pearsonema* sp.	1	0.02 (0–0.06)
Cestodes		
* Dipylidium caninum*	12	0.26 (0.11–0.4)
Taeniidae	7	0.15 (0.04–0.26)
* Spirometra* sp.	2	0.04 (0–0.1)
Trematodes		
* Alaria* sp.	1	0.02 (0–0.06)
* Heterobilharzia americana*	1	0.02 (0–0.06)
* Nanophyetus salmincola*	1	0.02 (0–0.06)
* Paragonimus kellicotti*	1	0.02 (0–0.06)
Protozoans		
Flagellates		
* Giardia* sp.	391	8.33 (7.54–9.12)
Coccidians		
* Cystoisospora ohioensis*-complex	104	2.22 (1.80–2.64)
* Cystoisospora* sp.	55	1.17 (0.86–1.48)
* Cystoisospora canis*	45	0.96 (0.68–1.24)
* Sarcocystis* sp.	16	0.34 (0.17–0.51)
* Cryptosporidium* sp.	13	0.28 (0.13–0.43)
* Neospora caninum*/*Hammondia*	3	0.06 (0–0.14)

^a^Original results reported as 263 samples positives for *Ancylostoma* sp. and 1 sample positive for *Uncinaria stenocephala*

The number of test results per laboratory and state ranged from 161 to 990, with a median of 469.20 ± 312.04 per laboratory, and 521.33 ± 312.04 per state. As shown in Table 3, significant differences were observed based on state-level geographic location of laboratories (*p* < 0.05). A significantly higher occurrence of GI parasites was observed in samples from New York (36.06%, 357/990), followed by Georgia (27.12% (48/177), and Alabama (23.36%; 36/161) (*p* < 0.05). Differences among the diagnostic method performed can also be noticed across laboratories (Table 3). Only the New York laboratory presented combined results of zinc sulfate (1.18 spg) and sucrose (1.33 spg) flotation methods for all samples. In addition, the overall occurrence was compared by month (Table 4). Significant differences were observed over the 1-year period (*p* < 0.05) with the highest percentages of positive samples recorded in May and June (25.67%; 124/483 and 24.95%; 118/473).

**Table 3:** Comparison of the endoparasite prevalence between nine US states, and the diagnostic method(s) used

State	Total (n)	Positives (n)	Prevalence % (95% CI)	Fecal flotation method
New York	990	357	36.06 (33.07–39.05)^a^	DC (S + ZS)
Pennsylvania	909	71	7.81 (6.07–9.56)^f^	SC (ZS)
Ohio	765	150	19.61 (16.79–22.42)^c, d, e^	DC (S)
Kansas	599	100	16.69 (13.71–19.68)^c, d, e^	DC (S or ZS)
Texas	464	94	20.26 (20.68–29.45)^b, e^	SC (S or ZS)
Virginia	366	79	21.58 (17.37–25.80)^b, d^	SC (S or ZS)
California	261	41	15.71 (11.29–20.12)^c, d, e^	DC (ZS)
Georgia	177	48	27.12 (20.57–33.67)^b^	DC (S or ZS)
Alabama	161	36	23.36 (15.92–28.80)^b, c^	DC (S or ZS or SN)

Prevalence with the same letter indicates no statistically significant difference (*p* > 0.05)

*DC* double centrifugation, *SC* single centrifugation, *S* Sheather’s sucrose, *ZS* zinc sulfate, *SN* sodium nitrate, *S + ZS* combined results of zinc sulfate and Sheather’s sucrose flotation for all samples

**Table 4:** Comparison of the prevalence of endoparasites in canine fecal samples between months

State	Total (*n*)	Positives (*n*)	Prevalence (%)
January	348	66	18.97 (14.85–23.08)^c, d^
February	344	71	20.64 (16.36–24.92)^a, d^
March	384	72	18.75 (14.85–22.65)^c, d^
April	452	91	20.13 (16.44–23.83)^b, c, d^
May	483	124	25.67 (21.78–29.57)^a^
June	473	118	24.95 (21.05–28.85)^a, b^
July	451	88	19.51 (15.85–23.17)^c, d^
August	443	88	19.86 (16.15–23.58)^b, c, d^
September	357	60	16.81 (12.93–20.69)^d^
October	386	70	18.13 (14.29–21.98)^c, d^
November	338	73	21.60 (17.21–25.98)^a, d^
December	233	55	23.61 (18.15–29.06)^a, c^

Prevalence with the same letter indicates no statistically significant difference (*p* > 0.05)

Table 5 illustrates the occurrence of endoparasites in dogs with respect to origin, breed, age, sex, and reproductive status. Based on these results, most parasite infections were diagnosed in puppies less than 1 year of age. Of the 1177 samples analyzed from dogs less than 1 year of age, 403 (34.24%) were positive for at least one parasite. Significant differences among Ancylostomatidae, *T*. *canis*, and *T*. *vulpis* infections were also observed among the different age groups (*p* < 0.05). *Toxocara canis* (7.22%; 85/1177) and Ancylostomatidae (6.71%; 79/1177) were more commonly detected in dogs under one year old. Among adult dogs (1–6 years), Ancylostomatidae (6.28%; 105/1671) and *T*. *vulpis* (2.57%; 43/1671) infections were more prevalent. We also observed a significantly higher occurrence of infection in hound dogs (37.7%; 216/573) than in other breed groups. As shown in Table 5, no significant differences were found in endoparasite infections between males and females (*p* > 0.05). In contrast, a significant difference was observed between intact and castrated/neutered dogs (*p* < 0.05). However, these results may be also impacted by age and not correlated with reproductive status alone, as most of the intact dogs are under one year old. A significant difference was also noticed in relation to the origin of samples (*p* < 0.05). A high occurrence of one or more parasite species identified in fecal samples analyzed from shelter dogs (53.98%; 122/226) was observed, as compared to a much lower occurrence in client-owned dogs (17.04%; 556/3262).

**Table 5:** Comparison of the prevalence of endoparasite infection by origin, breed, age, sex, and reproductive status

Variable	Total (n)	Positives (n)	Prevalence % (95% CI)
Origin			
Teaching hospital^a^	2819	406	14.40 (13.11–15.70)^a^
Outside practitioner^a^	443	150	33.86 (29.45–38.27)^b^
Research	375	135	36.00 (31.14–40.86)^b^
Shelter	226	122	53.98 (47.48–60.48)
Referral laboratory	35	6	17.14 (4.66–29.63)^a^
Other/Unknown	794	157	19.77 (17–22.54)
Breed group			
Mixed	1490	272	18.26 (16.29–20.22)^b^
Hound	573	216	37.70 (33.73–41.66)^a^
Sporting	648	114	17.59 (14.66–20.52)^b^
Herding	461	92	19.96 (16.31–23.61)^b^
Working	384	70	18.23 (14.37–22.09)^b^
Toy	387	43	11.11 (7.98–14.24)^c^
Non-sporting	251	36	14.34 (10.01–18.68)^b, c^
Terrier	179	24	13.41 (8.24–18.40)^b, c^
Foundation stock service	30	4	13.33 (1.17–25.50)^b, c^
Unknown	289	105	36.33 (30.79–41.88)
Age			
< 1 year (young)	1177	403	34.24 (31.53–36.95)
1–6 years (adult)	1671	249	14.90 (13.19–16.61)
> 6 years (senior)	1337	97	7.26 (5.86–8.65)
Unknown	507	227	44.77 (40.44–49.10)
Sex			
Male	2261	449	19.86 (18.21–21.50)
Female	1998	377	18.87 (17.15–20.58)
Unknown	433	150	34.64 (30.16–39.12)
Reproductive Status			
Male, intact	1098	308	28.05 (25.39–30.71)^a^
Male, castrated	1159	141	12.17 (10.28–14.05)^b^
Female, intact	800	231	28.88 (25.73–32.02)^a^
Female, spayed	1174	143	12.18 (10.31–14.05)^b^
Unknown	461	153	33.19 (28.89–37.49)

Prevalence with the same letter indicates no statistically significant difference (*p* > 0.05)

^a^Canine fecal samples originating from teaching hospitals and outside practitioners are considered client-owned (3262; 69.52%)

Additional comparisons between overall infections by protozoans and helminths can be found in Additional file 1: Tables S1 and S2.

## Discussion

Canine endoparasites were a common finding in fecal samples processed in the parasitology diagnostic laboratories in the USA included in this study, with 20.8% of dogs positive for at least one species, ranging from 7.81 to 36.6% across laboratories (Table 3). This wide occurrence range was likely influenced by characteristics of the sampled population, including geographic location and climate factors, and the various centrifugal flotation protocols and flotation solutions used. In addition, such factors could also have influenced the statistical analyses and other outcomes, including occurrence of positive dogs in each state and the different variables analyzed (e.g., Table 5). The majority of fecal samples analyzed originated from client-owned dogs presented to veterinary teaching hospitals (69.52%; 3,262/4692), which could have added some bias to our results. These dogs were presumed to be well cared for and assumed to receive appropriate veterinary attention, however, no information on antiparasitic chemoprophylaxis or routine diagnostic were available to substantiate this claim. Not surprisingly, samples from shelter dogs had a significantly higher occurrence of overall endoparasite infections (53.98%; Table 5), including protozoan and helminth infections specifically when compared to dogs with other origins (Additional file 1: Table S1). Shelter dogs were most likely parasitized due to a lack or lower frequency of anthelmintic treatment and increased presence of risk factors associated with transmission, including close contact with infected animals, environmental contamination, and possibly nutritional and immunosuppressive factors [18]. Our retrospective study also showed that breed had a significant effect on the prevalence of endoparasites. Overall, endoparasite infections were more frequent in hound breed dogs with an occurrence of 37.7% (216/573). Protozoans were the most common parasite group detected in hound dogs than in other breed groups (Additional file 1: Table S1). However, the different flotation protocols used across diagnostic laboratories may have influenced the occurrence rate between breed groups, geographic location, level of health care, behavioral predispositions, and lifestyle/amount of outdoor activity. Moreover, our results demonstrated no significant effect of sex on the occurrence of canine endoparasites (Table 5).

The overall prevalence of canine GI parasites in the USA reported from previous surveys is relatively consistent, ranging from 8.79 to 20.7% [5, 19, 20]. The occurrence of the most common endoparasites found in the present study is slightly higher but comparable to the 2018 prevalence data from the Companion Animal Parasite Council (CAPC) [12] (Table 6). In a one-year retrospective study, Little et al. [19] recorded a prevalence of 12.5% for GI parasites in dogs presenting to veterinary clinics in the USA. Mohamed et al. [20] reported a prevalence of 8.79% for intestinal nematode parasitism among pet dogs examined in veterinary hospitals from 2003 to 2006. In contrast, our results showed a similar occurrence rate of intestinal parasites to those recently reported by Stafford et al. [5] in dog parks from 30 major metropolitan areas. Similarly, occurrence of single or co-infections of intestinal parasites were detected in 24% [2] and 27% [21] of park-attending dogs from southern states. The apparent variability in canine endoparasite rates between previous studies and the present study could be related to the dog population considered in the survey, diagnostic method used, climatic and demographic factors, and/or different prevention methods adopted by the owners. Regarding co-infections, dogs infected with two or more species (3.82%; 179/4692) were less frequently identified than those with single infections (16.99%; 797/4692), similar to the findings of previous studies [19, 22].

**Table 6:** Comparative endoparasite prevalence data from the Companion Animal Parasite Council and the present retrospective study

State	*Giardia* sp. (%)		Ancylostomatidae (%)		*Toxocara canis* (%)		*Trichuris vulpis* (%)	
	Present study	CAPC 2018	Present study	CAPC 2018	Present study	CAPC 2018	Present study	CAPC 2018
Overall	8.33	6.44	5.61	2.96	2.49	1.89	2.43	0.67
New York	20.1	6.86	7.27	2.29	2.73	1.83	4.95	0.58
Pennsylvania	2.42	5.69	1.87	3.13	1.76	2.65	1.32	0.99
Ohio	6.67	5.17	7.06	3.20	2.61	2.69	1.96	1.32
Kansas	5.51	4.97	3.84	2.71	3.51	1.45	1.67	0.81
Texas	3.66	4.60	7.76	4.63	1.94	1.35	1.08	0.66
Virginia	8.74	4.25	7.92	3.83	2.46	1.70	1.64	0.95
California	8.81	9.90	NA	0.69	0.77	2.14	0.38	0.24
Georgia	2.82	3.90	11.86	3.84	4.52	1.80	8.47	0.96
Alabama	5.59	3.18	6.83	5.66	3.11	2.09	0.62	0.89

In our study, the overall occurrence of nematodes was 9.87%, which is higher than the prevalence rate of 3.49% reported by the CAPC [12], and 2.1% by a recent retrospective study from Oklahoma [22]. Ancylostomatidae was detected in all age classes (5.63%; 264/4692) but with higher frequency in dogs under 6 years old, corroborating findings of Little et al. [19] and Nagamori et al. [22]. In general, hookworm infections are often detected in both young and adult animals as dogs remain susceptible even in adulthood [23, 24]. Recent studies have revealed a gradual increase in hookworm prevalence in the USA over the past 10 years [22, 25]. This previous data along with the higher occurrence of Ancylostomatidae observed in our study may indicate the occurrence of anthelmintic resistance in the canine population. Presence of multiple drug resistance in *A. caninum* in the USA has been demonstrated by Jimenez Castro et al. [26] and is a significant emerging problem in the dog population. Moreover, the higher occurrence of Ancylostomatidae could have implications for public health, as the canine hookworm *A. caninum* can cause cutaneous larva migrans infection in humans [12].

*Toxocara canis* is another important zoonotic nematode detected in our study with an occurrence of 2.49% (117/4692). Unsurprisingly, considering its biology, *T. canis* infections seem to be influenced by age. The occurrence of *T. canis* in samples from young dogs was significantly higher than in those from adult dogs (*p* < 0.05). Higher *T. canis* prevalence in young animals often occurs due to immature immune system, and vertical transmission, primarily the transplacental route. Similar prevalence rates have been found in other studies, and are equally associated with younger dogs [19, 22]. A significant difference related to reproductive status was also noticed. Intact animals were more frequently infected by *T. canis* than castrated ones. However, this difference may be associated with age because most of the intact dogs analyzed in our study were less than one year old. *Toxocara canis* infections in dogs have public health significance due to the potential of this parasite to infect and cause several clinical syndromes in humans. Farmer et al. [27] estimated that over 16 million individuals (5.1%) in the USA may be exposed to or infected by *Toxocara* spp. These results emphasize the veterinarian’s important role in implementing appropriate control measures against endoparasites to prevent the spread of zoonotic parasites.

Canine whipworm, *T. vulpis*, was found in 2.43% (114/4692) of samples, corroborating results from Nagamori et al. [22]. As observed in the present study, adult dogs were more commonly infected by *T. vulpis* than young and senior dogs (*p* < 0.05). The overall occurrence between states ranged from 0.38% in California to 8.47% in Georgia. This difference could be associated with the sample origin and time of collection since peak infection usually occurs in the winter [25]. Seasonality of whipworm infections in dogs has been described by Drake and Carey [25] which also demonstrated a slight decrease in the prevalence of *T. vulpis* in dogs in the USA within the last decade. Recent studies combining classical microscopy-based centrifugal flotation methods and coproantigen ELISA revealed that centrifugal flotation alone tends to underestimate the prevalence of *Giardia*, *A. caninum*, *T. canis*, and *T. vulpis* [5, 19], and therefore it is likely that our study also underestimated their prevalence.

The low occurrence of tapeworms found in the study (Table 1) may be due to the fact that fecal flotation is not the most sensitive test for diagnosing infections with *Dipylidium* or Taeniidae-type eggs of *Taenia* and *Echinococcus* [28]. Tapeworm infection in companion animals in the USA is fairly common, but it is also largely underdiagnosed by fecal examination or even visual examination of feces for the presence of proglottids [29]. Adolph et al. [13] demonstrated that cestode infections were detected in less than 20% of truly infected dogs when centrifugal fecal flotation techniques were used as the diagnostic method. Therefore, this data allows us to suggest that the true prevalence of cestode infections in companion animals is underestimated by at least 80% using passive and centrifugal flotation. Canine tapeworm infections can be controlled by cestocidal anthelmintics (e.g., praziquantel, epsiprantel) associated with management practices to reduce the risk of re-exposure and reinfection. The use of such control measures would be expected to decrease the prevalence and prevent the transmission of cestodes to other hosts. Similarly, trematode eggs were a rare finding in our study with an occurrence of 0.02% (1/4692) for each of the following: *Alaria* sp., *H.* *americana*, *N. salmincola*, and *P.* *kellicotti*. The low detection of trematode species could also be correlated with the diagnostic techniques evaluated in this study as fecal sedimentation is the most accurate microscopy-based diagnostic test for detecting trematode eggs [28]. Geographic location could also be responsible for the low incidence in the present study, and the true overall USA prevalence of these trematode infections is likely higher.

Additional nematode species, including the zoonotic threadworm, *S.* *stercoralis*, and the lungworms *Crenosoma* sp. and *E. aerophilus*, were found at a low occurrence in this study. Generally, these parasites are not found at high prevalence in client-owned dogs in the USA [19, 22, 30]. Among the classical diagnostic tests, the Baermann test, rather than centrifugal fecal flotation, is usually recommended for detection of nematodes that shed larvae rather than eggs in feces (e.g., *S. stercoralis* and *Crenosoma* sp.) [28, 31]. Larvae may be observed in fecal flotations but are often damaged, and generally require a Baermann for diagnostic confirmation [28]. It is important to stress that *S.* *stercoralis* is likely underdiagnosed and is an important zoonotic parasite that infects dogs and cats as suggested by Barratt et al. [32] and Wulcan et al. [33]. Therefore, the correct use of preventive products in dogs is crucial for the control of endoparasites and could impact the likelihood of human infection. There are several single and combination active products approved for the treatment of roundworm, whipworm, and hookworm infections in dogs in the USA, including broad-spectrum heartworm preventives [12, 34].

Among non-zoonotic nematodes found in the study were species associated with the GI tract (*Physaloptera* sp., *U. stenocephala*, and *T. leonina*), and the respiratory tract (*E. boehmi*), which are occasionally reported at low prevalence in dogs from different regions of the United States [22, 29, 32]. In addition, the diagnosis of *Pearsonema* eggs in a single dog was likely due to contamination of the fecal sample with urine, as this capillarid genus is associated with the urinary bladder [29].

With respect to protozoan infections, *Giardia* sp. and *Cystoisospora* spp. were the most frequently observed parasites in the present study. *Giardia* infections have an important role in public health due to their zoonotic potential [7, 10, 35]. New York presented a higher occurrence of protozoan infection (26.87%; 266/990) compared with other states (Additional file 1: Table S2). However, this fact seems to be correlated with the diagnostic method employed (i.e., double centrifugation with zinc sulfate). The *Giardia* occurrence recorded in the present study was higher compared to the CAPC [12] estimates from pet dogs across the USA in the same time period (Table 6). Although no drugs are approved for treatment of *Giardia* infections in the USA, multiple-day dosing of fenbendazole alone or in combination with praziquantel and pyrantel has been shown to be effective [36–40]. Metronidazole-based treatment has also been commonly used in humans and dogs to decrease *Giardia* cyst excretion [41–44]. The commercial availability of metronidazole formulations varies from country to country. So far, there are no veterinary licensed metronidazole-based products available in the USA to treat canine giardiasis, however, human formulations have been used as extra-label therapies by veterinarians [42]. The CAPC [12] recommends centrifugal flotation with zinc sulfate as first-line diagnostic tests for protozoan infections. With suspected *Giardia* infections, a specific and sensitive fecal ELISA for antigen should also be included in the diagnostic examination, however, we should consider the occurrence of false-positive results [12, 45, 46].

Our study found an infection rate of 4.35% (204/4692) of *Cystoisospora* spp., with occurrence in young and adult dogs being significantly higher than in senior dogs. This rate is comparable to the study conducted by Little et al. [19] who reported a prevalence of 4.4% for *Cystoisospora* spp. in pet dogs, mainly in the West and Midwest regions of the USA. In addition, both Little et al. [19] and Nagamori et al. [22] found that *Cystoisospora* infections were significantly more common in dogs younger than 6 months of age, which supports the importance of veterinary care for dogs in the first year of life. With regard to treatment, despite several anticoccidial drugs and drug combinations reported for treating *Cystoisospora* infections, only sulfadimethoxine is label approved in the USA for treatment of bacterial enteritis associated with coccidiosis [12].

Our results emphasize that canine endoparasites of veterinary and public health importance are relatively prevalent despite current recommendations for routine diagnostics and the use of readily available broad-spectrum, effective, and safe anthelmintic products. One must keep in mind that some helminths and protozoan species may not be impacted by broad-spectrum products and could require additional treatment and diagnostic methods for identification. The current CAPC [12] guidelines recommend that every dog should be on a broad-spectrum parasite prevention protocol that provides year-round protection against GI helminths (particularly important for zoonotic parasites), in addition to macrocyclic lactone-based chemoprophylaxis against heartworm disease [47]. Nevertheless, only approximately one-third of dogs in the USA receive routine heartworm prevention, which may or may not treat infections by GI nematodes or tapeworms [48]. Therefore, it is important to emphasize that heartworm preventive products do not necessarily reduce or eliminate endoparasite infections among pet dogs. Veterinarians should be familiar with label claims for each product on the market to better educate their clients about what parasites are covered by these preventive products, especially GI nematodes and cestodes. Veterinarians should continue to emphasize the need for routine fecal screening, at least annually, even in dogs receiving appropriate heartworm prevention, and if parasites are noted, dogs must receive appropriate treatment.

## Conclusion

In summary, canine endoparasites are a common finding in fecal samples processed in parasitology diagnostic laboratories across the USA. The vast majority of samples came from client-owned animals that visited academic teaching hospitals or private veterinary practices. Various species of protozoans, nematodes, trematodes, and cestodes were diagnosed across different laboratories and states, including potentially zoonotic parasites. Our results highlight the importance of implementing broad-spectrum endoparasite prevention protocols coupled with routine fecal screening and treatment of any detected parasites, as well as pet owner education regarding parasite prevention, and anthelmintic regimens to reduce the risks of environmental contamination, re-infections, and zoonotic transmission.

## Supplementary Information

**Additional file 1**: **Table S1**. Prevalence comparison of protozoan and helminth infections by origin, breed, age, sex, and reproductive status. **Table S2**. Comparison of the prevalence of protozoan and helminth infections in dogs between nine US states.

## Data Availability

All data generated or analyzed during this study are included in this published article and its supplementary information files.

## Abbreviations

GI: Gastrointestinal
CAPC: Companion Animal Parasite Council
ELISA: Enzyme-linked immunosorbent assay
