# Supplementary material for: Retrospective study of canine endoparasites diagnosed by fecal flotation methods analyzed across veterinary parasitology diagnostic laboratories, United States, 2018
Source: Parasit Vectors. 2021 Aug 31;14:439. doi: 10.1186/s13071-021-04960-7 (PMC8406898; doi:10.1186/s13071-021-04960-7)
Supplement: Supplementary file 1 — Additional file 1: Table S1. Prevalence comparison of protozoan and helminth infections by origin, breed, age, sex, and reproductive status. Table S2. Comparison of the prevalence of protozoan and helminth infections in dogs between nine US states. [file 13071_2021_4960_MOESM1_ESM.docx]

**Table S1.** Prevalence comparison of protozoan and helminth infections by origin, breed, age, sex and reproductive status.

| Variable | Total number | Number of positive dogs (%, 95% CI) | |
| --- | --- | --- | --- |
|  |  | **Protozoan infection** | **Helminth infection** |
| Origin |  |  |  |
| Teaching Hospital | 2,819 | 222 (7.88, 6.88-8.87) ^b^ | 220 (7.8, 6.81-8.79) ^c^ |
| Outside Practitioner | 443 | 86 (19.41, 15.73-23.1) ^c^ | 83 (18.74, 15.1-22.37) ^b^ |
| Research | 375 | 119 (31.73, 27.02-36.44) ^a^ | 16 (4.27, 2.22-6.31) ^d^ |
| Shelter | 226 | 78 (34.51, 28.32-40.71) ^a^ | 67 (29.65, 23.69-35.6) ^a^ |
| Referral Laboratory | 35 | 2 (5.71, 0-13.4) ^b^ | 4 (11.43, 0.89-21.97) ^b, c, d^ |
| Other/Unknown | 794 | 76 (9.57, 7.53-11.62) | 97 (12.22, 9.94-14.49) |
| Breed Group |  |  |  |
| Mixed | 1,490 | 152 (10.2, 8.66-11.74) ^b^ | 148 (9.93, 8.41-11.45) ^b, c, d^ |
| Hound | 573 | 135 (23.56, 20.09-27.03) ^a^ | 87 (15.18, 12.24-18.12) ^a^ |
| Sporting | 648 | 75 (11.57, 9.11-14.04) ^b^ | 50 (7.72, 5.66-9.77) ^d, f^ |
| Herding | 461 | 54 (11.71, 8.78-14.65) ^b^ | 53 (11.5, 8.58-14.41) ^a, c^ |
| Working | 384 | 34 (8.85, 6.01-11.7) ^b^ | 46 (11.98, 8.73-15.23) ^a, b^ |
| Toy | 387 | 33 (8.53, 5.74-11.31) ^b^ | 11 (2.84, 1.19-4.5) ^e, f^ |
| Non-sporting | 251 | 26 (10.36, 6.59-14.13) ^b^ | 14 (5.58, 2.74-8.42) ^d^ |
| Terrier | 179 | 13 (7.26, 3.46-11.06) ^b^ | 11 (6.15, 2.63-9.66) ^d, e^ |
| Foundation Stock Service | 30 | 1 (3.33, 0-9.76) ^b^ | 3 (10, 0-20.74) ^a, d^ |
| Unknown | 289 | 60 (20.76, 30.79-41.88) | 64 (22.15, 17.36-26.93) |
| Age |  |  |  |
| <1 year (young) | 1,177 | 281 (23.87, 21.44-26.31) ^a^ | 175 (14.87, 12.84-16.9) ^a^ |
| 1 – 6 years (adult) | 1,671 | 107 (6.4, 5.23-7.58) ^b^ | 161 (9.63, 8.22-11.05) ^b^ |
| >6 years (senior) | 1,337 | 34 (2.54, 1.7-3.39) ^c^ | 66 (4.94, (3.78-6.1) ^c^ |
| Unknown | 507 | 161 (31.76, 27.7-35.81) | 85 (16.77, 13.51-20.02) |
| Sex |  |  |  |
| Male | 2,261 | 279 (12.34, 10.98-13.7) ^a^ | 209 (9.24, 8.05-10.44) ^a^ |
| Female | 1,998 | 207 (10.36, 9.02-11.7) ^a^ | 201 (10.06, 8.74-11.38 ^a^ |
| Unknown | 433 | 97 (22.4, 18.47-26.33) | 77 (17.78, 14.18-21.38) |
| Reproductive Status |  |  |  |
| Male, Intact | 1,098 | 216 (19.67, 17.32-22.02) ^a^ | 119 (10.84, 9-12.68) ^a^ |
| Male, Castrated | 1,159 | 63 (5.44, 4.13-6.74) ^b^ | 90 (7.77, 6.22-9.31) ^c^ |
| Female, Intact | 800 | 137 (17.13, 14.51-19.74) ^a^ | 112 (14, 11.6-16.4) ^b^ |
| Female, Spayed | 1,174 | 67 (5.71, 4.38-7.03) ^b^ | 87 (7.41, 5.91-8.91) ^c^ |
| Unknown | 461 | 100 (21.69, 17.93-25.45) | 79 (17.14, 13.7-20.58) |

Prevalence with the same letter indicates no statistically significant difference (*p* > 0.05)

**Table S2**. Comparison of the prevalence of protozoan and helminth infections in dogs between nine US states.

| **State** | **Total number** | **Number of positive dogs (%, 95% CI)** | |
| --- | --- | --- | --- |
|  |  | **Protozoan infection** | **Helminth infection** |
| New York | 990 | 266 (26.87, 24.11-29.63) ^a^ | 134 (13.54, 11.4-15.67) ^b^ |
| Ohio | 765 | 71 (9.28, 7.22-11.34) ^d^ | 94 (12.29, 9.96-14.61) ^b^ |
| Kansas | 599 | 51 (8.51, 6.28-10.75) ^d^ | 53 (8.85, 6.57-11.12) ^c, d^ |
| Texas | 464 | 43 (9.27, 6.63-11.91) ^c, d^ | 59 (12.72, 9.68-15.75) ^b^ |
| Virginia | 366 | 53 (14.48, 10.88-18.09) ^b^ | 41 (11.20, 7.97-14.43) ^b, c^ |
| Pennsylvania | 909 | 27 (2.97, 1.87-4.07) ^e^ | 46 (5.06, 3.64-6.49) ^e^ |
| Georgia | 177 | 16 (9.04, 4.82-13.26) ^b, d^ | 36 (20.34, 14.41-26.27) ^a^ |
| California | 261 | 36 (13.79, 9.61-17.98) ^b, c^ | 6 (2.30, 0.48-4.12) ^e^ |
| Alabama | 161 | 20 (12.42, 7.33-17.52) ^b, d^ | 18 (11.18, 6.31-16.05) ^b, d^ |

Prevalence with the same letter indicates no statistically significant difference (*p* > 0.05)
